# 

**Published:** 1930

**Authors:** 


### The Medical Library of the University of Bristol,

WITH WHICH IS INCORPORATED

**The Library of the Bristol Medico-Chirurgical Society.**

---

*The following donations have been received since the publication of the list in October,* 1930.

*December*, 1930.

| | | |
|---|---|---|
| Dr. Erik Agduhr (1) .. .. .. .. | 1 | volume |
| Hamilton Bailey, Esq., F.R.C.S. (2) .. .. | 1 | ,, |
| Dr. R. J. A. Berry (3) .. .. .. .. | 3 | volumes |
| British Medical Association (4) .. .. .. | 1 | volume |
| Miss A. Ruth Fry (5) .. .. .. .. | 2 | volumes |
| Glasgow Royal Maternity and Women's Hospital (6) | 1 | volume |
| Professor E. W. Hey Groves, M.S. (7) .. .. | 5 | volumes |
| London School of Hygiene and Tropical Medicine (8) | 1 | volume |
| Michell Clarke Memorial Fund (9) .. .. | 1 | ,, |
| Royal College of Surgeons of England (10) .. | 1 | ,, |
| Dr. Frank A. Stahl (11) .. .. .. .. | 1 | ,, |
| Dr. George H. Weaver (12) .. .. .. | 1 | ,, |

Unbound periodicals have also been received from Professor Hey Groves.

### THE ONE HUNDRED AND THIRTY-FOURTH LIST OF BOOKS.

---

The figures in round brackets refer to the figures after the names of the donors. The books to which no such figures are attached have either been bought from the Library Fund or received through the *Journal.*

| | | | |
|---|---|---|---|
| **Agduhr, E., and Stenström, N.** | *The Appearance of the Electrocardiogram in Heart Lesions produced by Cod Liver Oil Treatment* .. .. .. .. .. .. .. | (1) | 1930 |
| **Bailey, H.** .. .. .. | *Emergency Surgery,* Vol. I. .. .. .. .. | (2) | 1930 |
| **Bedingfield, H.** .. .. | *Visceroptosis and Allied Abdominal Conditions associated with Chronic Invalidism* .. .. | | 1930 |
| **Berry, R. J. A.** .. .. | *Regional Anatomy,* 3 Vols. .. .. .. .. | (3) | 1902 |
| **Bland-Sutton, Sir J.** | *On Faith and Science in Surgery* .. .. | | 1930 |
| *Clinical Interpretation of Aids to Diagnosis.* Vol. I. .. .. .. | | | 1930 |
| **Crookshank, F. G.** .. | *Individual Diagnosis* .. .. .. .. .. .. | | 1930 |
| **Davidson, M.** .. .. | *Cancer of the Lung* .. .. .. .. .. .. .. | | 1930 |
| **Davis, G. G.** .. .. | *Applied Anatomy* .. .. .. 8th Ed. | (7) | 1929 |
| **Douglas, W. C.** .. .. | *Elements of Medical High Frequency and Diathermy* .. .. .. .. .. .. .. .. .. | | 1930 |
| *Essays and Addresses.* | By a Surgeon .. .. .. .. .. .. .. .. | | 1930 |
| **Ewing, A. W. G.** .. | *Aphasia in Children* .. .. .. .. .. .. | | 1930 |

| | | |
|---|---|---|
| **Fraser, J.** .. .. .. | *The Breast in Health and Disease* .. (7) | 1929 |
| **Gloyne, S. R.** .. .. | *Clinical Pathology of Thoracic Puncture Fluids* .. .. .. .. .. .. .. .. (7) | 1930 |
| **Headridge, D., and Gibson, S. K.** | *Dental Anatomy—Human and Comparative* .. .. .. .. .. .. .. .. .. | 1928 |
| **Helmont, J. B. van** | *Oriatrike* .. .. .. .. .. .. .. .. .. (9) | 1662 |
| **Howard, C.** .. .. | *After Consulting Hours* .. .. .. .. .. .. | 1930 |
| **Kanga, B. S.** .. .. | *Handbook of Tuberculosis* .. .. .. (7) | 1930 |
| **McKenzie, D.** .. .. | *Diathermy Medical and Surgical in Oto-Laryngology* .. .. .. .. .. .. .. .. .. | 1930 |
| **McKittrick, L. S., and Root, H. F.** | *Diabetic Surgery* .. .. .. (7) | 1929 |
| **Monrad-Krohn, G. H.** | *The Clinical Examination of the Nervous System* .. .. .. .. .. .. .. 5th Ed. | 1930 |
| **Osler, Sir W.** .. .. | *Bibliotheca Osleriana* .. .. .. .. .. .. | 1929 |
| **Paterson** .. .. .. | *Sick Children* .. .. .. .. .. .. .. .. .. | 1930 |
| *Report on the Physical Welfare of Mothers and Children.* | Vols. 3 and 4 (Carnegie United Kingdom Trust) .. (5) | 1917 |
| **Stahl, F. A.** .. .. | *Concerning the Origin and Development of the Chorion, Amnion and Yolk Sac* .. (11) | 1930 |
| **Tidy, H. L.** .. .. | *Synopsis of Medicine* .. .. .. 5th Ed. | 1930 |
| **Toomey, N.** .. .. | *Diseases of the Skin* .. .. .. .. .. .. | 1930 |
| **Watson-Williams, P.** | *Chronic Nasal Sinusitis and its Relation to General Medicine* .. .. .. .. .. .. .. .. | 1930 |
| **Weaver, G. H.** .. .. | *John Haygarth* .. .. .. .. .. .. .. .. (12) | 1930 |

TRANSACTIONS, REPORTS, JOURNALS, ETC.

| | |
|---|---|
| British Medical Association Annual Handbook, 1930–31 .. .. (4) | 1930 |
| Episcopal Hospital Reports, Philadelphia. Vol. VI., 1921–30 .. .. | 1930 |
| Glasgow Royal Maternity and Women's Hospital Report, 1929 .. (6) | 1930 |
| London School of Hygiene and Tropical Medicine. Collected Addresses. Vol. V., 1928–29 .. .. .. .. .. .. .. .. .. .. .. .. .. .. .. (8) | 1930 |
| Royal College of Surgeons of England Calendar, 1930 .. .. .. .. .. | 1930 |
| Surgical Clinics of North America, October, 1930 .. .. .. .. .. .. | 1930 |

### Local Medical Notes.

EXAMINATION RESULTS.

**University of Bristol.**—Students of the University have recently passed the following examinations :—

F.R.C.S.—J. T. Chesterman, A. C. Fisher, K. H. Pridie.

M.D.—T. A. Brand.

M.B., Ch.B.—*Supplementary First Examination. Pass :* P. N. Heron. *In Physics and Biology only,* G. W. Vowles.

M.B., Ch.B. — *Final Examination. (Part I.). Pass (including Forensic Medicine and Toxicology) :* G. T. Bevir, R. N. O'D. Burns, R. G. C. G. Carlson, A. M. Davies, G. M.

Evans, A. L. Eyre-Brook (with distinction in Materia Medica, Pharmacy, Pharmacology and Therapeutics, Pathology and Forensic Medicine and Toxicology), F. E. Fletcher (with distinction in Forensic Medicine and Toxicology), J. D. Hughes, H. James, J. Ridgway, Frances N. Salisbury (with distinction in Pathology), O. E. L. Sampson (with distinction in Materia Medica, Pharmacy, Pharmacology and Therapeutics, and Forensic Medicine and Toxicology), J. P. P. Stock (with distinction in Pathology). *Part I. only :* J. R. Gibbs.

M.B., CH.B.—*Final Examination.* (*Part II.*). *Pass in Group I. only :* Eveline M. D. M. Collinson, Rowena M. Hickman.

L.D.S.—*Second Professional Examination. Pass* (*In Dental Mechanics and Dental Metallurgy* (*Completing Examination*) *:* R. E. W. Ellis-Smith. *In Dental Materia Medica* (*Completing Examination*) *:* Helena C. L. Blinkworth, W. V. Whiteford. *In Dental Materia Medica only :* G. N. Minifie.

L.D.S.—*Final Professional Examination. Pass :* E. R. Carpenter.

CONJOINT BOARD. — M.R.C.S., L.R.C.P. — *Pass* (*In Chemistry :* W. H. Hayes, Gwyneth M. Griffiths, Eileen R. Atchison. *In Physics :* W. H. Hayes, Eileen R. Atchison. *In Anatomy and Physiology :* A. F. Fowler. *In Physiology :* R. H. Moodie. *In Pharmacology and Materia Medica :* N. C. Coombs, J. F. H. Eagles. *In Pathology :* J. L. S. James. *In Medicine :* A. A. G. Flemming,* H. M. Strover,* K. I. Nicholls,* R. P. Lucas,* U. M. Hopkins.* *In Surgery :* R. P. Lucas,* Margaret E. Williams,* W. Woolley, R. G. Wilbond, L. H. Griffiths,* F. T. J. Hobday, J. L. S. Coulter, R. H. Moore, J. A. Kersley. *In Midwifery :* Anne E. Williams-James, R. P. Lucas,* Margaret E. Williams,* L. R. Jordan, Ida B. Saxby, J. L. S. Coulter, R. G. Wilbond, R. H. Moore, J. A. Kersley, O. J. Bollon, W. A. F. Taylor, T. J. Ashley, Elizabeth S. G. Owen, L. C. Holland.

L.D.S., R.C.S.—*First Professional Examination. Part I.* (*Dental Mechanics*) *:* F. S. Bromley, N. G. Harrison, A. E. Hawkins. *Part II.* (*Dental Metallurgy*) *:* F. S. Bromley, Beryl M. Davies, Margaret D. Hooper, J. S. Cochrane, W. N. Trays. *Part III.*(*a*). (*Anatomy and Physiology*) *:* A. C. Davies, S. A. McCandlish, N. E. Miles, H. F. Vere. *Part III.*(*b*). (*Dental Anatomy and Physiology*) *:* P. J. F. Shepherd.

* Qualified.

### Index.

Anthropology.—Keith, Sir A. The Beddoe Memorial Lecture : Anthropology, old and new, 287.
Ashton, L. P.—A case of von Recklinghausen's Disease (multiple neurofibromata) with spontaneous fractures, 219.
Auricular fibrillation, Stokes-Adams Syndrome produced by digitalis in a case of.—T. F. R. Hewer, 135.

Beddoe Memorial Lecture ; Anthropology, old and new.—Sir A. Keith, 287.
Berry, R. J. A. :—
  Hospitals — Voluntary or self-supporting ? 19.
  Child Guidance, II.—Clinics from the Brain Physiologist's Viewpoint, 120.
Blachford, J. V.—Observations on Epilepsy, 169.
Bodman, F. and Bodman, P.—Perforation in Paratyphoid B, 317.
Books, Reviews of—
  Agduhr, E. and Stenström, N.—The appearance of the electrocardiogram in heart lesions produced by cod liver oil treatment, 338.
  Anæsthesia and anæsthetics. — F. S. Rood and H. N. Webber, 156
  Aphasia in children.—A. W. G. Ewing, 335.
  Army Medical Corps, A Short History of.—F. Smith, 240.
  Bailey, H.—Physical Signs in Clinical Surgery, 155.
  Basu, B. D.—Diabetes Mellitus, 150.
  Bedingfield, H.—Visceroptosis and Allied Abdominal Conditions, 242.
  Berry, R. J. A.—Through the States with a Seeing Eye, 69.
  Bland-Sutton, Sir J.—On Faith and Science in Surgery, 239.
  Brockbank, E. M.—Diagnosis and Treatment of Heart Disease, 240.
  Burdett's Hospitals and Charities, 75.
  Cancer, Colour and.—C. E. Iredell, 337.
  Cancer of the Larynx.—Sir St. C. Thomson, 335.
  Cancer of the Lung and other Intrathoracic Tumours. — M. Davidson, 333.

Books, Reviews of (continued)—
  Cancer and Radium (Curietherapy).—D. C. L. Fitzwilliams, 153.
  Catarrh, Nasal.—W. S. Low, 155.
  Chest, Percussion of. — J. B. MacDougall, 150.
  Children, Aphasia in.—A. W. G. Ewing, 330.
  Children, Sick, Diagnosis and Treatment.—D. Paterson, 333.
  Clinical Interpretation of Aids to Diagnosis, Vol. 1., 334.
  Coli Bacilluria, Colporrhaphy and Rheumatism, Recent work on.—E. H. Roberts, 74.
  Colporrhaphy, Rheumatism and Coli Bacilluria, Recent work on.—E. H. Roberts, 74.
  Cosmetic Treatment of Skin Complaints.—E. Kromayer, 152.
  Crookshank, F. G. — Individual Diagnosis, 332.
  Curietherapy — Radium and Cancer. — D. C. L. Fitzwilliams, 153.
  Davidson, M.—Cancer of the Lung and other Intrathoracic Tumours, 333.
  Diabetes Mellitus.—B. D. Basu, 150.
  Diagnosis, Aids to, Clinical Interpretation of, 334.
  Diagnosis, Individual. — F. G. Crookshank, 332.
  Diathermy, The Elements of High Frequency and. — W. C. Douglass, 244.
  Diathermy, Medical and Surgical, in Oto-Laryngology.—D. MacKenzie, 242.
  Diseases of Sick Children, Nursing and.—A. Moncrieff, 245.
  Doctor's Job, The.—A. Lloyd-Williams, 149.
  Douglass, W. C.—The Elements of Medical High Frequency and Diathermy, 244.
  Electrocardiogram in Heart Lesions Produced by Cod Liver Oil Treatment.—E. Agduhr and N. Stenström, 338.
  Essays and Addresses.—A Surgeon, 246.
  Ewing, A. W. G.—Aphasia in Children, 335.
  FitzSimon, F. W.—The Snake Park, 157.

Z

Books, Reviews of (continued)—
FitzSimon, F. W.—Snake Venoms, 157.
Fitzwilliams, D. C. L.—Radium and Cancer (Curietherapy), 153.
Fowler, Sir J. K.—The Sthenics, 69.
Gibson, A. G.—Mycoses of the Spleen, 245.
Gordon, R. G. and Thomson, F. G. —The Physiological Principles of Hydrology, 243.
Hæmodynamics.—P. B. Kittel, 72.
Heart Disease, Diagnosis and Treatment of.—E. M. Brockbank, 240.
Heart Lesions, The Appearance of the Electrocardiogram in, Produced by Cod Liver Oil Treatment.—E. Agduhr and N. Stenström, 338.
Hewer, E. E. and Sandes, G. M.—Introduction to the Study of the Nervous System, 71.
Heymans, C.—*Le Sinus Carotidien et les autres zones Vasosensibles Réflexogènes*, 70.
Hickman, Henry Hill, Centenary Exhibition, 331.
Hospitals and Charities, Burdett's, 75.
Howard, C.—After Consulting Hours, 331.
Hutton, I. E.—The Hygiene of Marriage, 75.
Hydrology, The Physiological Principles of.—R. G. Gordon and F. G. Thomson, 243.
Infectious Diseases, Acute.—J. D. Rolleston, 71.
Iredell, C. E.—Colour and Cancer, 337.
Jones, Sir R.—Orthopedic Surgery, 73.
Kittel, P. B.—Hæmodynamics, 72.
Kromayer, E.—The Cosmetic Treatment of Skin Complaints, 152.
Larkin, A. J.—Radium in General Practice, 154.
Larynx, Cancer of.—Sir St. C. Thomson, 335.
Lloyd-Williams, A.—The Doctor's Job, 149.
Love, R. J. M.—A Shorter Surgery, 149.
Low, W. S.—Nasal Catarrh, 155.
Lung, Cancer of.—M. Davidson, 333.
McConnel, J. K.—Shorter Convalescence, 243.
MacDougall, J. B.—Percussion of the Chest, 150.

Books, Reviews of (continued)—
MacKenzie, Sir C.—The Action of Muscles, 241.
MacKenzie, D.—Diathermy, Medical and Surgical, in Oto-Laryngology, 242.
Marriage, The Hygiene of.—I. E. Hutton, 75.
Martindale and Westcott.—The Extra Pharmacopœia, Vol. II., 156.
Medical Annual, 157.
Medicine, A Synopsis of.—H. L. Tidy, 332.
Medico - Legal Problems.—Lord Riddell, 152.
Moncrieff, A.—Nursing and Diseases of Sick Children, 245.
Monrad-Krohn, G. H.—Clinical Examination of the Central Nervous System, 241.
Morphine Habit and its Painless Treatment.—G. L. Scott, 153.
Muscles, The Action of.—Sir C. MacKenzie, 241.
Nasal Catarrh.—W. S. Low, 155.
Nasal Sinusitis.—P. Watson-Williams, 246.
Nervous System, Clinical Examination of.—G. H. Monrad-Krohn, 241.
Nursing and Diseases of Sick Children.—A. Moncrieff, 245.
Orthopedic Surgery.—Sir R. Jones, 73.
Oto-Laryngology, Diathermy in.—D. MacKenzie, 242.
Paterson, D.—Sick Children, 333.
Percussion of the Chest.—J. B. MacDougall, 150.
Pharmacopœia.—Martindale and Westcott, Vol. II., 156.
Physical Signs in Clinical Surgery. —H. Bailey, 155.
Physiology, Applied.—S. Wright, 70.
Radium and Cancer (Curietherapy). —D. C. L. FitzWilliams, 153.
Radium in General Practice.—A. J. Larkin, 154.
Rheumatism, Colporrhaphy and Coli Bacilluria, Recent Work on. —E. H. Roberts, 74.
Riddell, Lord.—Medico - Legal Problems, 152.
Roberts, E. H.—Recent Work on Colporrhaphy, Rheumatism and Coli Bacilluria, 74.
Rolleston, J. D.—Acute Infectious Diseases, 71.

BOOKS, REVIEWS OF (continued)—
Rood, F. S. and H. N. Webber.—Anæsthesia and Anæsthetics, 156.
Sandes, G. M. and Hewer, E. E. — Introduction to the Study of the Nervous System, 71.
Scott, G. L.—The Morphine Habit, 153.
Sechehaye, A.—The Treatment of Tuberculosis with Umckaloabo, 151.
Sinusitis, Nasal. — P. Watson-Williams, 246.
Skin Complaints, Cosmetic Treatment of.—E. Kromayer, 152.
Skin Diseases, The Treatment of. —N. Toomey, 334.
Smith, F.—A Short History of the Army Medical Corps, 240.
Snake Park, The.—F. W. FitzSimons, 157.
Snake Venoms.—F. W. FitzSimons, 157.
Spleen, Mycoses of.—A. G. Gibson, 245.
Stenström, N. and E. Agduhr.—The Appearance of the Electrocardiogram in Heart Lesions Produced by Cod Liver Oil Treatment, 338.
Sthenics, The.—Sir J. K. Fowler, 69.
Surgery, Physical Signs in Clinical. —H. Bailey, 155.
Surgery, The Dramatic in.—G. G. Taylor, 155.
Surgery, A Shorter.—R. J. M. Love, 149.
Taylor, G. G.—The Dramatic in Surgery, 155.
Testicular Grafting from Ape to Man.—S. Voronoff, 72.
Thomson, E. G. and R. G. Gordon. —The Physiological Principles of Hydrology, 243.
Thomson, Sir St. C.—Cancer of the Larynx, 335.
Tidy, H. L.—A Synopsis of Medicine, 332.
Toomey, N.—The Treatment of Skin Diseases, 334.
Treves-Barber, T. H.—Varicose Veins, 73.
Tuberculosis, Treatment with Umckaloabo. — A. Sechehaye, 151.
Tumours, Intrathoracic, and Cancer of the Lung. — M. Davidson, 333.

BOOKS, REVIEWS OF (continued)—
Umckaloabo, The Treatment of Tuberculosis with. — A. Sechehaye, 151.
Varicose Veins.—T. H. Treves-Barber, 73.
Visceroptosis and Allied Abdominal Conditions. — H. Bedingfield, 242.
Voronoff, S.—Testicular Grafting from Ape to Man, 72.
Ward, E.—General Practice, 241.
Watson-Williams, P. — Chronic Nasal Sinusitis, 246.
Webber, H. N. and F. S. Rood.—Anæsthesia and Anæsthetics, 156.
Wright, S.—Applied Physiology, 70.
Bridges, Dr. Robert—Obituary, 159.
Bristol Hospitals Council, 339.
Bristol Medical Dramatic Club, 88.
Bristol Medico-Chirurgical Society, 78, 165, 346.
British Association for the Advancement of Science. Annual Meeting in Bristol, 248.
Burden, Rev. H. N.—Obituary, 249.
Bush, J. Paul.—Obituary, 342.

Cancer, Experiences in Radium Treatment of.—A. R. Short, 82.
Cancer of the Tonsils.—E. Watson-Williams, 78.
Carter, T. M.—Obituary, 164.
Child Guidance, I. Work in New York.—Mrs. Posthuma, 111.
Child Guidance, II. Clinics from the Brain Physiologist's Viewpoint.—R. J. A. Berry, 120.
Child Guidance, III. Work in Great Britain.—R. G. Gordon, 126.
Cooke, R. V.—Five Cases of Injury to Main Vessels Occurring in Civil Practice, 311.

Dental Infections.—G. F. Fawn, 137.
Dental Research.—R. H. McKeag, 143.
Digitalis, Stokes-Adams Syndrome Produced by.—T. F. R. Hewer, 135.
Dyspepsia.—A. T. Todd, 83.

Eclampsia.—G. H. Temple, 307.
Embolism and Thrombosis.—W. A. Owen, 29.
Epilepsy.—J. V. Blachford, 169.

Exophthalmic Goitre.—A. R. Short, 185.

Fawn, G. F.—Dental Infections, 137.
Food.—Nixon, J. A.—Influence of Food on the Production and Prevention of Disease. Long Fox Memorial Lecture, 255.

Gordon, R. G.—Child Guidance, III. Work in Great Britain, 126.
Graves' Disease.—C. E. K. Herapath, 193.

Harvey Memorial Fund, 77.
Herapath, C. E. K.—The Treatment of Graves' Disease, 193.
Hewer, T. F. R.—Stokes-Adams Syndrome produced by Digitalis, 135.
Hewer, T. F. R.—Changes in the Spleen in Acute Pyogenic Infections, 197.
Hospitals :—
Bristol Hospitals Council, 339.
Hospital Amalgamation, 340.
Hospitals — Voluntary or Self-supporting.—R. J. A. Berry, 19.

Intestinal Obstruction.—D. P. D. Wilkie, 97.

Jordan, L. R.—Tuberculosis of the Tracheo-Bronchial Lymph Nodes of the Lung, 225.

Keith, Sir A.—Beddoe Memorial Lecture : Anthropology Old and New, 287.

Local Medical Notes, 87, 167, 254, 351.
Long Fox Memorial Lecture : The Influence of Food on the Production and Prevention of Disease.—J. A. Nixon, 255.

McKeag, R. H.—Recent Dental Research, 143.
Medical Library of the University of Bristol, 84, 166, 252, 350.

Nixon, J. A.—Long Fox Memorial Lecture : The Influence of Food on the Production and Prevention of Disease, 255.
Noble, J. I.—Typhoid Fever with Co-existent Bacillus Coli Infection, 323.

Obituaries—
Dr. Robert Bridges, 159.
Rev. H. N. Burden, 249.
J. Paul Bush, 342.
T. M. Carter, 164.
Owen, A. W.—The Infective Factor in Thrombosis and Embolism, 29.

Paratyphoid B, Perforation in.—F. Bodman and P. Bodman, 317.
Posthuma, Mrs.—Child Guidance, I. Work in New York, 111.
Pyogenic Infections. — T. F. R. Hewer, 197.

Radium in the Treatment of Cancer.—A. R. Short, 82.
Recklinghausen's Disease. — L. P. Ashton, 219.

Short, A. R.—Radium in the Treatment of Cancer, 82.
Short, A. R. — Radium in Exophthalmic Goitre, 185.
Spleen, in Acute Pyogenic Infections.—T. F. R. Hewer, 197.
Stokes-Adams Syndrome Produced by Digitalis.—T. F. R. Hewer, 135.

Temple, G. H. — Treatment of Eclampsia, 307.
Thrombosis and Embolism.—A. W. Owen, 29.
Todd, A. T.—Dyspepsia, 83.
Tonsil, Cancer of. — E. Watson Williams, 78.
Tracheo-Bronchial Lymph Nodes of the Lung.—L. R. Jordan, 225.
Tuberculosis of the Tracheo-Bronchial Lymph Nodes of the Lung.—L. R. Jordan, 225.
Typhoid Fever with Co-existent Bacillus Coli Infection.—J. I. Noble, 323.

Walters, C. F.—The Duty of Medicine to the Race, 1.
Watson-Williams, E.—Cancer of the Tonsils, 78.
Wilkie, D. P. D.—Acute Intestinal Obstruction, 97.
Winford Orthopædic Hospital, Opening of, 161.

J. W. ARROWSMITH LTD., BRISTOL

### Publications Received.

From THE ACTINIC PRESS LTD.:

*Therapeutic Uses of Infra-Red Rays.* By W. A. TROUP, M.B.

From BAILLIÈRE, TINDALL & COX:

*Gleanings from General Practice.* By G. ASHTON, R.A.M.C.

From CASSELL & CO. LTD.:

*The Students' Handbook of Surgical Operations.* 5th Edition. By SIR F. TREVES, BART., G.C.V.O., C.B., LL.D., F.R.C.S. Revised by C. P. G. WAKELEY, F.R.C.S. (Eng.), F.R.S. (Edin.).

From GALE & POLDEN LTD.:

*R.A.M.C. Aids to Dispensing.* By G. ASHTON, R.A.M.C.

From THE LANCET LTD.:

*Clinical Interpretations of Aids to Diagnosis.* Vol. I.

From H. K. LEWIS & CO. LTD.:

*Stepping Stones to Surgery: Anatomy Applied to Surgery.* By L. B. RAWLING, M.B., Ch.B., (Cantab), F.R.C.S.

*Technique and Results of Grafting Skin.* By H. K. CHRISTIE, M.S. (N.Z.), F.R.C.S. (Eng.).

*The Treatment of Asthma.* By A. H. DOUTHWAITE, M.D., F.R.C.P. (Lond.).

*Colour and Cancer: An Investigation.* By C. E. IREDELL, M.D. (Lond.), M.R.C.P.

*Diet and Care of the Surgical Case.* By R. H. BOYD, M.B., CH.B. (N.Z.), F.R.C.S. (Edin.)

From JOHN WRIGHT & SONS LTD.:

*Emergency Surgery.* Vol. I. By HAMILTON BAILEY, F.R.C.S.

*British Journal of Surgery.* Vol. XVIII., No. 70, 1930.

*A Synopsis of Medicine.* 5th Edition. By H. L. TIDY, M.A., M.D., B.CH. (OXON.), F.R.C.P. (Lond.).

*A B C of Child Health.* By A. WEAR, C.M.G., M.D., B.S., D.P.H.

